# Epicatechin Influence on Biochemical Modification of Human Erythrocyte Metabolism and Membrane Integrity

**DOI:** 10.3390/ijms252413481

**Published:** 2024-12-16

**Authors:** Annamaria Russo, Giuseppe Tancredi Patanè, Giuseppina Laganà, Santa Cirmi, Silvana Ficarra, Davide Barreca, Elena Giunta, Ester Tellone, Stefano Putaggio

**Affiliations:** 1 Department of Chemical, Biological, Pharmaceutical and Environmental Sciences, University of Messina, Viale Ferdinando Stagno d’Alcontres 31, 98166 Messina, Italy; arusso@unime.it (A.R.); giuseppe.patane@studenti.unime.it (G.T.P.); santa.cirmi@unime.it (S.C.); sficarra@unime.it (S.F.); ester.tellone@unime.it (E.T.); stefano.putaggio@studenti.unime.it (S.P.); 2 Virology and Microbiology AOOR Papardo-Piemonte, Viale Ferdinando Stagno d’Alcontres, 98166 Messina, Italy; elenagiunta@live.it

**Keywords:** red blood cells, epicatechin, antioxidant systems, Band-3 protein, anion exchange, erythrocyte metabolism

## Abstract

Red blood cells (RBCs) are the main cells of the blood, perform numerous functions within the body and are in continuous contact with endogenous and exogenous molecules. In this context, the study aims to investigate the effect of epicatechin (EC) (flavan-3-ols) on the erythrocytes, analyzing the protective effect of the molecule and the action exerted on metabolism and RBC membrane. The effect of EC on RBC viability has been evaluated through the change in hemolysis and methemoglobin, assessing caspase 3 activity and performing a cytofluorometric analysis. Next, the impact of the molecule on RBC metabolism was assessed by measuring anion flux kinetics, ATP production, and phosphatase activity. Finally, an evaluation of the potential protection against different stressors was performed. Our results show no detrimental effects of EC on RBCs (no change in hemolysis or methemoglobin and no caspase 3 activation recorded); rather, a protective effect was recorded given the reduction in hemolysis induced by hydrogen peroxide treatment and temperature increase. The increase in anion exchange and intracellular ATP values, with the inhibition of phosphatase PTP1B activity, highlights several biochemical alterations induced by EC. The present results contribute to clarifying the influence of EC on RBCs, confirming the beneficial effects of catechins.

## 1. Introduction

**Figure 1:**
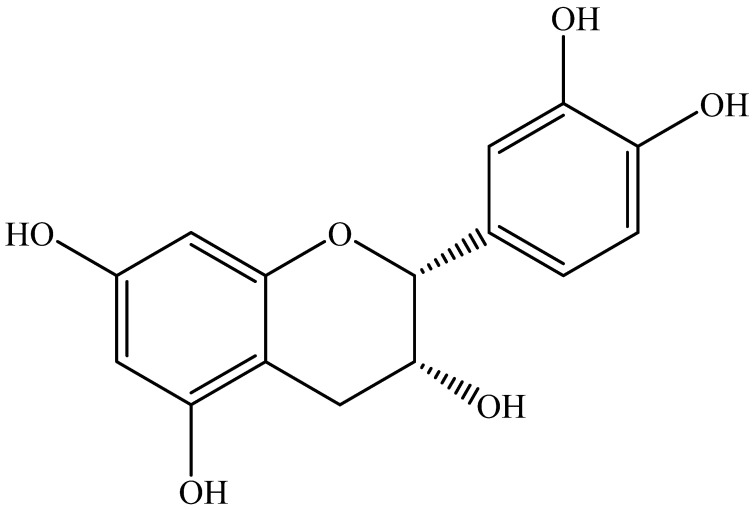
Epicatechin ((2*R*,3*R*)-2-(3,4-dihydroxyphenyl)-3,4-dihydro-2*H*-chromene-3,5,7-triol) chemical structure.

Catechins are molecules that belong to the class of flavonols, a subgroup of flavonoids (secondary metabolites of plants) and are present in numerous foods of plant origin such as fruit and vegetables, or in some types of tea and cocoa seeds such as *Camellia sinensis* and *Theobroma cacao* [1]. Chemically, catechins, and more generally flavan-3-ols, are characterized by two benzene rings (indicated by “A” and “B”) and by a hydropyran heterocyclic ring (indicated by “C”), which presents on the C-3 carbon a hydroxyl group (-OH). Benzene rings are rich in hydroxyl groups, and based on the placement of these on the “A” or “B” ring, it is possible to distinguish meta-dihydroxylated or meta-trihydroxylated compounds and monohydroxylated, orthohydroxylated or vicinal-hydroxylated compounds, respectively. By further analyzing this class of compounds, it is possible to recognize three chiral centers in the C2, C3 and C4 positions of the heterocyclic ring, allowing the identification of different stereoisomers [2]. Since in nature the R configuration is mainly observed, initially this was the only possible configuration assumed; then, further studies revealed the S configuration on C2 [3]. When the hydroxyls on C2 and C3 are oriented in the same direction, the prefix “epi” is used, distinguishing (+) catechin (2S,3R) and (−) epi (2R,3R) (see Figure 1) [4].

Given the different dietary sources of these compounds and their high consumption, catechins have aroused particular interest in the world of research, especially for the effect they have on health. Catechins have been demonstrated to reduce cardiovascular diseases, inhibit the oxidation of lipids and cholesterol, reducing the state of inflammation, have a preventive action in diabetes, and are involved in slowing down metabolic syndromes. In addition, they have a potential reno-protective, antihypertensive and neuroprotective action [2,5,6,7,8,9]. Gour et al., have also specifically shown that epicatechin (EC) reduces endothelial activation by modulating cell adhesion and nitric oxide bioavailability. In addition, EC exhibited anti-sickling activity mediated by the inhibition of hemoglobin polymerization and reducing red blood cell membrane fragility [10]. Since the effect of the molecule is closely linked to its cell targeting pathway, the purpose of this paper was to evaluate the effects of EC on cellular metabolism, using erythrocytes as a study model. Red blood cells (RBCs) represent the main component of human blood; they are responsible for transporting gases and nutrients throughout the body and taking away the waste products. They are used for the assessment of the individual’s health and for the study of various physiological and metabolic aspects. In addition, RBCs, being in direct contact with molecules of an exogenous and endogenous nature to which they react through structural, morphological and metabolic modifications, constitute a unique biochemical index [11].

RBCs have a biconcave disc shape and an average life span of 120 days [12]. They participate in various physiological functions that continuously expose them to oxidative stress (Fenton and Haber–Weiss reactions); moreover, they maintain the systemic acid–base balance, maintain cardiovascular balance thanks to the regulation of nitric oxide (NO) metabolism and adenosine triphosphate (ATP) synthesis, control blood rheology, and also have an erythrocrine function [13,14,15]. In addition, Pretorius et al. demonstrated that oxidative stress and the upregulation of proinflammatory molecules contribute to biochemical changes in the erythrocyte membrane, causing morphological alterations [16]. Therefore, RBCs represent a valid study model improved even by their metabolic simplicity due to the absence of a nucleus and organelles that would limit their metabolic pathways. In RBCs, the glucose-6-phosphate (G6P) catabolism is shifted through the pentose phosphate (PPP) pathway in the high oxygenation state (HOS) to generate the reducing cofactor NADPH, and through the glycolytic pathway in the low oxygenation state (LOS) with the generation of energy in the form of ATP and NADH [17]. The regulation of these metabolic fluxes is driven by the T and R conformational states of hemoglobin (Hb) through its binding with the N-terminal cytoplasmic domain of Band 3 protein (CDB3) [11]. Band 3 protein (B3, SLC4A1 or AE1 anion exchanger 1) is part of a membrane cluster of many proteins, including Hb, anhydrase carbonic II, and aquaporin 1, which promote the electroneutral Cl^−^/${HCO}_{3}^{-}$ exchange, intracellular pH, and modulation of RBC metabolism. To this metabolon, the ankyrin-spectrin-AE1 linkage provides an anchor to the erythrocyte membrane skeleton and RBC shape regulation [18,19,20]. CDB3 is a substrate of the apoptosis executioner caspase 3, responsible for triggering programmed cell death. In erythrocytes, caspase 3 activation is stimulated by acidification of the cytosolic environment and leads to catalytic cleavage of the AE1 cytosolic domain and degradation of cellular proteins [21]. In these cells, the regulation of intracellular pH (pH_i_) is modulated by NHE1, a member of a family of electroneutral exchangers ubiquitously expressed. NHE1 mediates the exchange of intracellular H^+^ with extracellular Na^+^, while ${HCO}_{3}^{-}$ exits from AE1 in exchange for Cl^−^. AE1 and NHE1 functionality in the control of pH_i_ are closely related, and the intracellular alkalinization state is a common feature of proliferative processes [22,23]. Matteucci et al. demonstrated that NHE1Na^+^/H^+^ exchanger is strongly inhibited by EC; the inhibition may alter pHi, and in this view, we have tried to evaluate the EC impact on other RBC proteins, including AE1, caspase 3, protein tyrosine phosphatase 1B (PTP-1B), and Hb [24]. Our approach fills some gaps in knowledge on EC modulation of cell metabolism and opens the way for further research to improve understanding of the clinical applicability of EC in diseases.

## 2. Results

Catechins, and, in particular, epicatechin, due to their high hydrophobicity, tend to accumulate at the level of the cell membrane. Therefore, the first step of the study was to evaluate the EC effect on RBC membrane integrity. At all concentrations tested (0–25–50–75–100 μM), EC did not cause significant damage, as the percentage of hemolysis was less than 3% even after 12 h of incubation. Similarly, the oxidative state of Hb did not appear altered in the presence of the molecule, since no increased values of methemoglobin were recorded in erythrocytes treated with EC (0–25–50–75–100 μM) for up to 12 h.

### 2.1. Epicatechin and Osmotic Fragility

**Figure 2:**
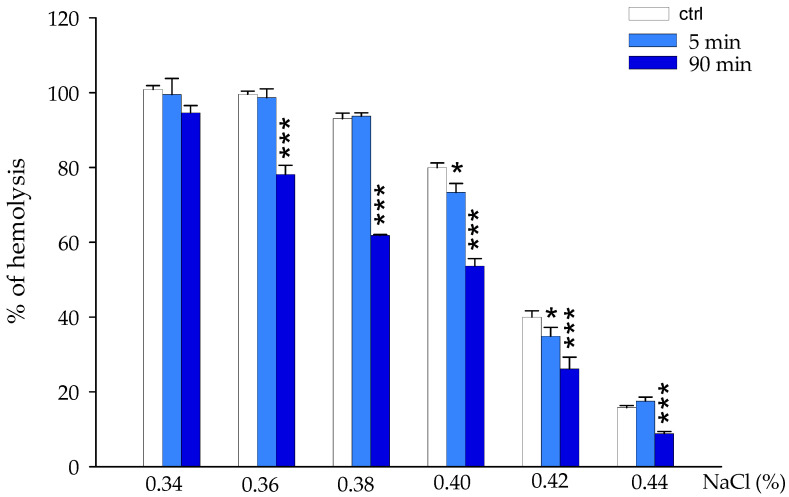
Effect of epicatechin 50 μM on erythrocyte membrane fragility after incubation with different NaCl concentrations (from 0.34 to 0.44%). RBCs in the absence of EC (white boxes), pretreated with 50 μM EC for 5 min (light blue boxes) and for 90 min (blue boxes). Hemolysis was measured by spectrophotometric absorbance of the supernatant at 576 nm. * *p* < 0.05 vs. ctrl, *** *p* < 0.001 vs. ctrl.

The EC accumulation at the membrane level could alter the biochemical-physical properties of the structure and affect the deformability of the RBCs; to test this hypothesis, the effect of EC on the membrane at different concentrations of NaCl was evaluated. The results in Figure 2 show that EC protects RBCs from osmotic hemolysis at all NaCl concentrations tested (from 0.34 to 0.44%). The protective effect becomes more evident after 90 min of incubation, suggesting the contact time between EC and erythrocytes as an important factor for the accumulation of the molecule on the membrane and for cellular protection. 

### 2.2. Heat-Induced Hemolysis

**Figure 3:**
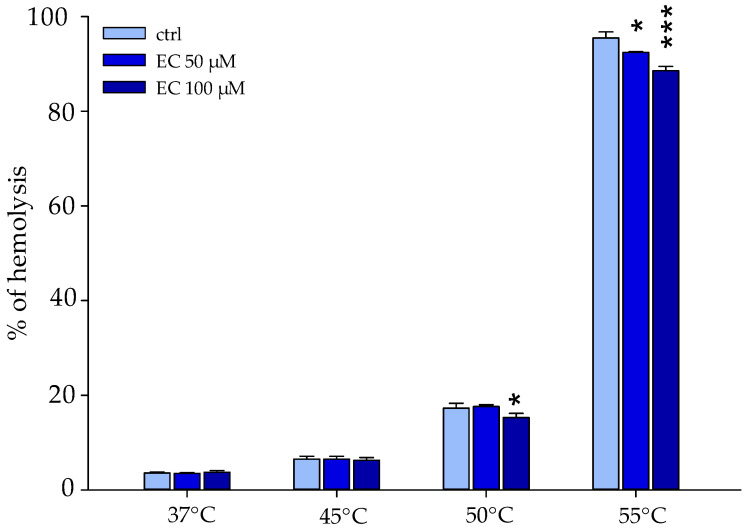
Protective effect against temperature increase exerted by EC on the erythrocyte membrane. RBCs in the absence of EC (light blue boxes), RBCs pretreated with 50 μM of EC for 30 min (blue boxes), RBCs pretreated with 100 μM of EC for 30 min (dark blue boxes). Absorbance value (at 576 nm). * *p* < 0.01 vs. ctrl, *** *p* < 0.001 vs. ctrl.

The effect of EC on membrane integrity as temperature changes was tested, since it is known that temperature can influence the elasticity of the RBC membrane and alter the deformation capacity of the cell [25]. The results of experiments performed after incubation of RBCs with EC (50 and 100 μΜ) at several temperatures (from 37 °C to 55 °C) for 30 min are shown in Figure 3. Interestingly, there is a slight but significant protection exerted by EC at a temperature of 50 °C, which becomes more evident at 55 °C.

### 2.3. Effect of Hydrogen Peroxide on Erythrocyte Membrane Integrity

**Figure 4:**
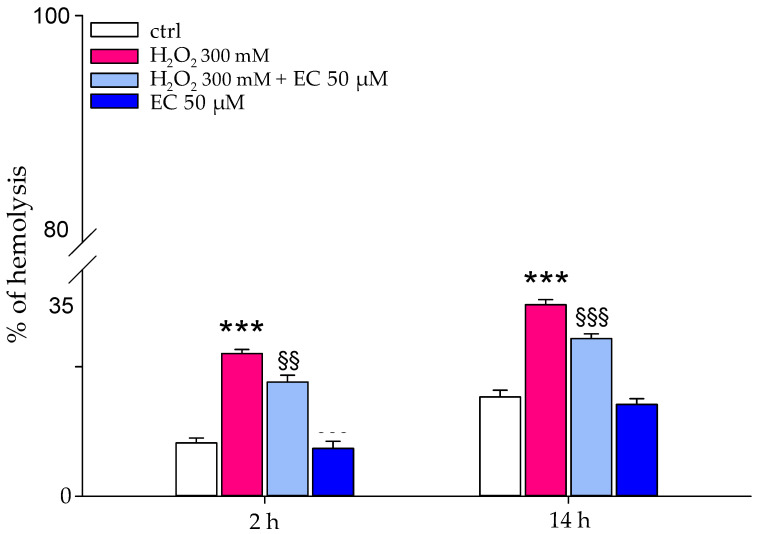
Protective effect of EC against H_2_O_2_ exposure for 2 and 14 h. RBCs in the absence of EC (white boxes); RBCs pretreated with EC 50 μM for 30 min and then exposed to 300 mM H_2_O_2_ for 2 h (red boxes); RBCs pretreated with EC 50 μM for 30 min and then exposed to 300 mM H_2_O_2_ for 14 h. Hemolysis was measured by spectrophotometric absorbance of the supernatant at 576 nm. *** *p* < 0.01 vs. ctrl, §§ *p* < 0.01 vs. H_2_O_2_ 300 mM, §§§ *p* < 0.001 vs. H_2_O_2_ 300 mM.

An evaluation of the protective capacities of EC on the RBC membrane was also carried out against a strong oxidizing agent such as hydrogen peroxide. RBCs incubated for 30 min with EC were exposed to H_2_O_2_ (300 mM) for 2 and 14 h, respectively. As shown in Figure 4, the presence of the molecule protects the cell from oxidant-induced hemolysis, reducing it by about 10%. The inhibiting power exerted by the EC did not change even with an increase in the exposure time (14 h) to H_2_O_2_.

### 2.4. Effect of EC on RBC Morphology

**Figure 5:**
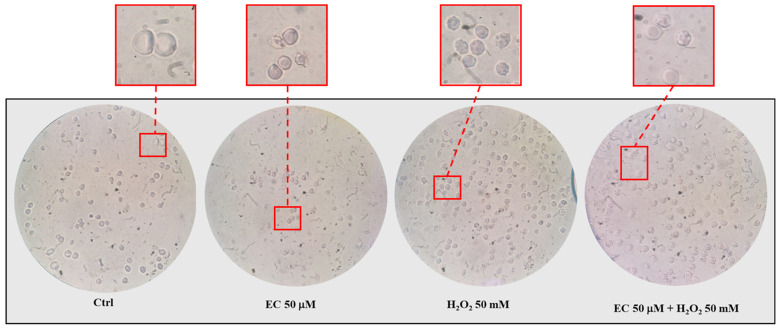
The figure shows the effects exerted on the RBC membrane by EC (50 μM) and H_2_O_2_ (50 mM) after 6 h of incubation. In detail, in the control (ctrl; RBC in the absence of EC and H_2_O_2_) it is possible to observe the classic biconcave disk shape typical of the cells. EC 50 μM (effect of EC on the RBC membrane): RBCs show a slight and negligible change in their structure compared to the control; H_2_O_2_ 50 mM (RBCs treated with H_2_O_2_ for 6 h): shrinkage and alteration of the erythrocyte membrane structure due to the action of the stressor agent can be observed; EC 50 μM + H_2_O_2_ 50 mM (RBCs pretreated with EC for 30 min, then incubation with H_2_O_2_): EC partially inhibited RBC alteration.

The morphology of RBCs was evaluated after incubation of cells with H_2_O_2_ (50 mM) and in the presence of EC (50 μM). The results are shown in Figure 5. In these conditions, a morphological alteration of the erythrocytes was evidenced by few spicules protruding from the surface of the cell membrane. This morphology, attributable to acanthocytes, was probably due to an alteration of lipids and membrane proteins caused by the oxidant. Alteration of RBCs was partially although not totally inhibited by the presence of EC (right quadrant).

### 2.5. Effect of Epicatechin on Anion Exchange

**Figure 6:**
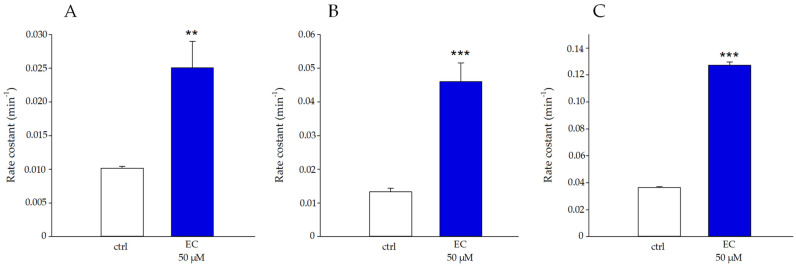
Sulphate flux measured in RBCs in the absence (white boxes) and presence of 50 μM EC (blue boxes), at temperature values of 20 °C (Section “(**A**)”), 30 °C (section “(**B**)”), and 40 °C (section “(**C**)”), respectively. ** *p* < 0.01 vs. ctrl, *** *p* < 0.001 vs. ctrl.

The effect of EC (50 μM) on the kinetics of the anionic exchanger at various temperatures (20, 30 and 40 °C) was tested. The results are shown in Figure 6. The EC acted on the kinetics of the flux, which increased at each temperature tested. In detail, at 20 and 30 °C, the value of the rate constant for the kinetics of the anion exchange was equal to about 0.010 min^−1^ in RBCs in the absence of EC, and for erythrocytes treated with EC (50 μM), the values were 0.025 and 0.046 min^−1^ at 20° and 30 °C, respectively. The kinetics increased by increasing the temperature to 40 °C; in this case, the rate constant values varied from 0.036 min^−1^ to 0.12 min^−1^ (respectively, in the absence and presence of EC 50 μM).

### 2.6. Influences of EC on Hb Structure

**Figure 7:**
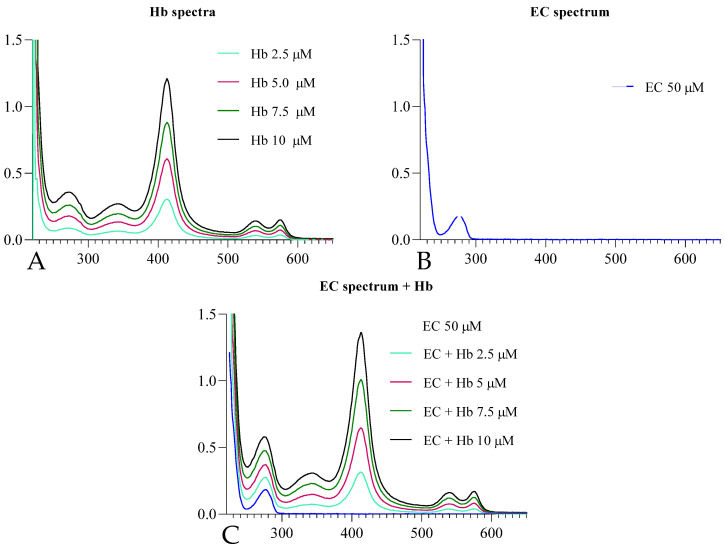
Section (**A**) shows the spectra of Hb from 2.5 μM to 10 μM; section (**B**) shows the spectrum of 50 μM epicatechin; section (**C**) shows the spectra resulting from titration of EC (50 μM) with Hb until the concentration of 10 μM is reached. As section (**C**) of the panel shows, there appears to be no interaction between EC and the protein given the absence of isosbestic dots and the lack of shift in the absorption bands.

The kinetics of AE1 are modulated by the interaction of Hb with CDB3 and by the phosphorylation state of this domain. To evaluate a potential interaction of the molecule with one of these modulatory mechanisms, the structural interaction of EC with Hb was spectrophotometrically tested. The results are shown in Figure 7. Significant Abs can be observed in the range of 253–300 nm, typical of EC. After the addition of increasing concentrations of Hb (0–10 μΜ), no changes in the shape and/or position of the Abs bands are observed, indicating the absence of a significant interaction between the EC and the protein.

### 2.7. EC Effect on Caspase 3

**Figure 8:**
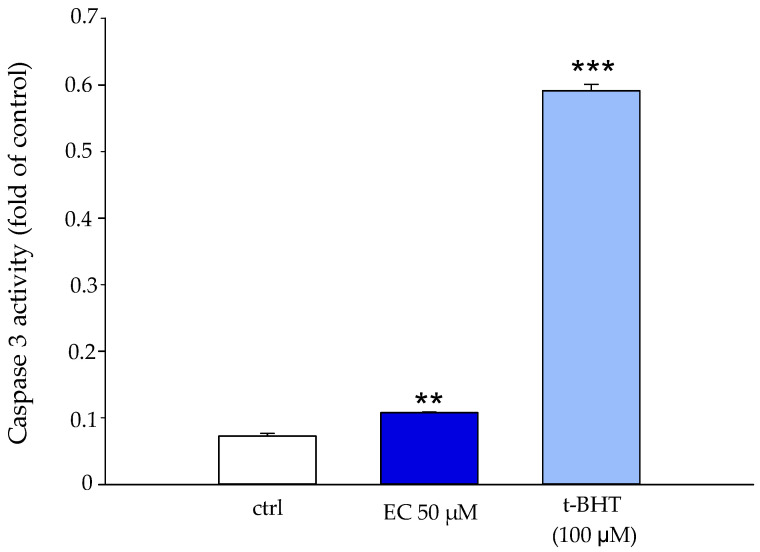
Caspase 3 activity in RBCs in the absence (white boxes) and presence of 50 μM EC (blue boxes) and in the presence of 100 μM t-BHT (light blue), respectively. ** *p* < 0.01 vs. ctrl, *** *p* < 0.001 vs. ctrl.

CDB3 is known to be a substrate of the apoptosis executioner caspase 3. The effect of EC was tested on the activation state of the protein, and the results are shown in Figure 8. The experiments were performed by incubating RBCs in the presence and absence of 50 μM EC for 30 min at 37 °C. The presence of EC (50 µM) caused a slight activation of caspase 3, which, although significant, appeared very low compared to the activation of t-BHT, a known activator of caspase 3.

### 2.8. Flow Cytometry

**Figure 9:**
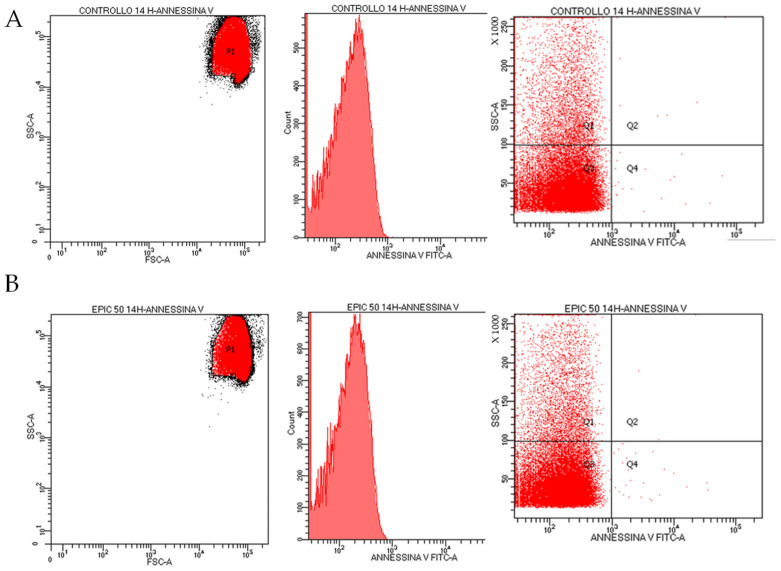
Cytofluorometry analysis of red blood cells incubated overnight in the absence (section (**A**)) and presence of 50 μM EC (section (**B**)).

Furthermore, the effect of epicatechin on cell viability was evaluated by cytofluorimetric analysis. Following incubation of the samples with ANNESSIN V, no externalization of phosphatidylserine was recorded. In Figure 9, results show that 50 μM EC does not cause damage to the erythrocyte membrane and does not alter the welfare status of erythrocytes. This result further confirms previous data on caspase 3 dormancy.

### 2.9. Influence of Epicatechin on PTP-1B Activity

**Figure 10:**
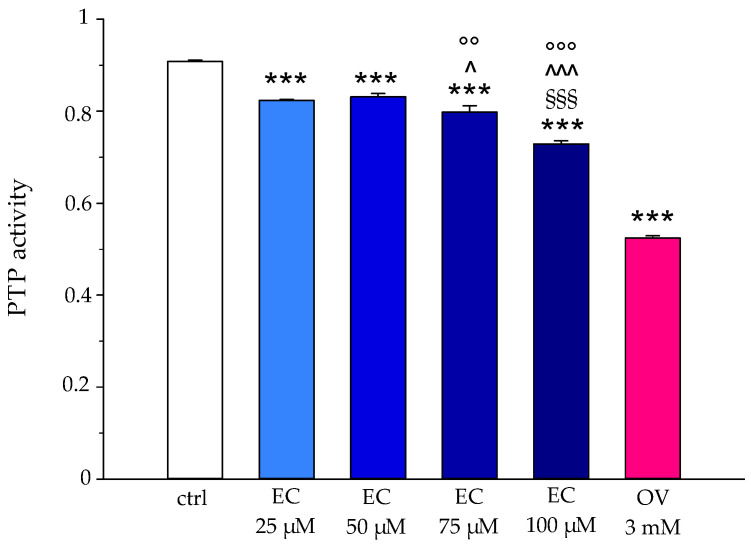
PTP-1B activity in human RBCs, in the absence (white box) and presence of 25–50–75–100 μM of EC (blue boxes), compared with the effect of 3 mM OV (red box). *** *p* < 0.001 vs. ctrl, §§§ *p* < 0.001 vs. EC 75 μM, ^ *p* < 0.05 vs. EC 25 μM, ^^^ *p* < 0.001 vs. EC 25 μM, °° *p* < 0.01 vs. EC 50 μM, °°° *p* < 0.001 vs. EC 50 μM.

The potential alteration of the phosphorylation state of CDB3 was evaluated by testing the activity of PTP-1B in the absence and presence of EC (0–25–50–75–100 μΜ) and comparing the effect with that of OV (3 mM), a known PTP-1B inhibitor. The results are shown in Figure 10, where it is possible to see a slight but significant inhibition of catalytic activity already at 25 μM, becoming more evident at 100 μM of EC [26,27].

### 2.10. Influence of Epicatechin on ATP Levels

**Figure 11:**
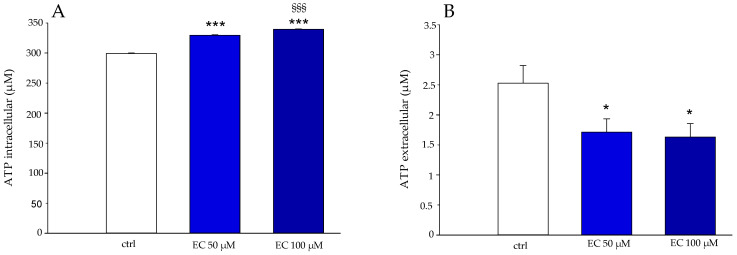
Effect of 50 and 100 μM epicatechin (blue boxes) on intracellular (**A**) and extracellular (**B**) ATP values in RBCs incubated for 30 min at 37 °C. RBCs in the absence of EC (white box). * *p* < 0.05 vs. ctrl, *** *p* < 0.001 vs. ctrl, §§§ *p* < 0.001 vs. EC 50 μM.

The action of EC (50 and 100 μΜ) was also evaluated on intracellular ATP levels and nucleotide release from erythrocytes. The results in Figure 11 show a slight but significant increase in intracellular ATP levels in RBCs incubated with EC for 30 min at 37 °C (Figure 11A). Figure 11B shows a decrease in levels of ATP released from RBCs following EC treatment under the same experimental conditions.

In detail, a light increase in intracellular ATP production, of about 10%, was observed in RBCs treated with EC 50 and 100 μM, while a reduction in extracellular ATP, of about 30%, was observed.

## 3. Discussion

The effect of epicatechin on red blood cells is expressed through various targets: in particular, the inhibition of osmotic fragility and the protection against hydrogen peroxide and heat suggest mechanical action of the molecule, potentially due to its accumulation on the membrane (see Figure 2, Figure 3, Figure 4 and Figure 5) [28]. EC seems to spread over the bilayer surface, forming hydrogen bonds with the head group part and the bilayer concurrently with a reduction in hydrogen bonds with water. Hydrogen bonds seem to be a key force in the adsorption of the polyphenol on the bilayer surface. The accumulation of EC at the membrane level could lead to a potential increase in erythrocyte volume and consequently to a greater resistance of the cells to external insults. As described by Ponder, RBCs are cells sensitive to the variation in the osmolarity of the solution, or to the variations in the chemical-physical parameters of the solution in which they are found. In hypotonic solutions, for example, erythrocytes swell and undergo lysis. Increasing the volume of the erythrocyte membrane can potentially reduce this phenomenon, thus increasing the resistance of RBCs [29]. Added to this action is the well-known antioxidant activity of catechins which is carried out by partially neutralizing the oxidizing action of hydrogen peroxide and limiting damage to the red blood cell membrane (see Figure 4 and Figure 5). The intensity of this activity is probably due not only to its individual potentiality, but also to its affinity for phospholipid bilayers mainly based on the 3’ and 4’ hydroxyl groups of the molecule.

The increase in anionic flux, induced in RBCs treated with EC (see Figure 6), could also be linked to the presence of the polyphenol in the phospholipid bilayer that potentially exerts its action directly on AE1. This increase in the rate of anionic flux also contributes to the elimination of CO_2_ produced by metabolically active tissues. In this context, the fast CO_2_ and peroxynitrite transport improves the antioxidant effect of EC, because it limits the generation of secondary free radical intermediates such as carbonate and nitrogen dioxide radicals that are harmful to the cell membrane. All these findings are in line with the protective effect we revealed about the absence of meta-Hb, no increase in hemolysis, no activation of caspase 3 and no apoptosis onset [30,31]. Moreover, our results indicate a slight, but significant, inhibition of EC-mediated PTP-1B activity in a concentration-dependent manner. This inhibition resulting in the increase in the phosphorylation state of CDB3 could contribute to anion exchange alteration as measured by us. The phosphorylation of AE1 primarily occurs at tyrosine 8 and 21, while secondary phosphorylation affects tyrosine 359 and 904 [32]. Campanella et al. reported that the phosphorylation of CDB3 tyrosine 21 reversibly causes the detachment and activation of GE with an increase in the glycolytic pathway and energy production (see Figure 11) [33]. The EC effect on PTP-1B can be explained by a direct action of EC-mediated PTP-1B and/or by a metabolic pathway in which the plasma membrane Ca^2+^-ATPase (PMCA) acts. Kuban-Jankowska et al. presented a computational analysis in breast cancer cells of the most predicted catechin binding to PTP-1B, which may decrease the enzymatic activity of PTP-1B phosphatase [34]. Rinaldi et al. demonstrated that catechin inhibitor intensity on PMCA activity is related to the orientation of -OH in C3 [35]. PMCA is the main regulator of intracellular Ca^2+^ levels in RBCs; its inhibition would lead to an increase in intracellular Ca^2+^, which may provide a stimulus for Ca^2+^-dependent calpain that in turn would cause PTP-1B inactivation through a proteolytic cleavage [36]. However, once again, our findings point towards a direct action of EC on PTP-1B because no activation of caspase 3 was revealed that would follow the increase in intracellular Ca^2+^. Furthermore, we measured an increase in intracellular ATP levels that would lead to increased PMCA activity, restoring intracellular Ca^2+^, which would suppress the calpains’ cleavage of PTP-1B 1B [37]. In addition, it has been shown that anionic flux is linked to the metabolic state of RBCs and that ATP deficiency reduces AE1 activity, due to reduced phosphorylation of AE1 amino acid residues. In this context, ATP could also act as a potential phosphate group donor, thus allowing AE1 phosphorylation. This is in line with our results showing an inhibition of phosphatase activity and an increase in intracellular ATP concentration [20].

PTP-1B is the key modulator of the insulin pathway and, specifically, it is a tyrosine phosphatase that inactivates the insulin cascade via dephosphorylation in tyrosine residues of the insulin receptor and IRS1 [38]. In a broader view, the inhibition of PTP-1B activity by EC, improving insulin sensitivity, can be positively related to diabetes conditions. In this context, EC might mitigate the hormone resistance by limiting the dephosphorylation effects along the insulin signal transduction pathway. In accordance with these findings, several studies demonstrated EC association with insulin sensitivity and glucose homeostasis. Further, considering the common links between the pathogeneses of diabetes mellitus and obesity, PTP-1B inhibitors could be a potential effective treatment for both diseases [39,40]. Confirming our assumptions, several studies show that EC also presents activity against different pathological conditions, such as inflammatory states, cancer, hypertension, and neurodegenerative diseases [41,42,43,44]. Finally, an in vivo study has shown that daily intake of EC (30–400 mg/day, for 3 months) leads to a decrease in the progression of pathological conditions and in the biomarkers of oxidative stress, confirming the antioxidant effect of the molecule [45]. Yamazaki et al. demonstrated that EC pretreatment for 2 or 10 days significantly reduced infarct size in rats 48 h after ischemia-reperfusion injury; in treated animals, significant reductions in tissue oxidative stress at the infarct region were observed [46].

## 4. Materials and Methods

### 4.1. Reagents and Compounds

The reagents used for the trial were purchased from Sigma Aldrich (St. Louis, MO, USA). Human blood was obtained from healthy donors, aged between 27 and 30 years. The erythrocytes were stored in tubes with ethylenediaminetetraacetic acid (EDTA) as an anticoagulant, and used fresh. The study was approved by a Local Ethics Committee (prot. 71–23 of 5 April 2023) in accordance with the Declaration of Helsinki. The stock solution of epicatechin was prepared with dimethyl sulfoxide (DMSO), and the different concentrations used in the experiments were obtained from it.

### 4.2. Preparation of Erythrocytes

Erythrocytes were collected with EDTA and washed three times with NaCl 0.9% (isosmotic solution). The blood was centrifuged at 3000 rpm for 5 min at 4 °C, and subsequently, the supernatant was removed from the erythrocytes. Subsequently, erythrocytes were suspended in a buffer, 2-[4-(2-hydroxyethyl) piperazin-1-yl]ethanesulfonic (HEPES), 20 mOsmol, pH 7.4; osmolarity was measured using the Omostat OM-6020 (Daiichikagakuco, Kyoto, Japan). The experiments were conducted with a hematocrit value of 3%. The degree of hemolysis and the levels of methemoglobin formed during incubation with different concentrations of epicatechin were recorded at the end of the incubation time, as reported in the literature by Tellone and collaborators [47].

### 4.3. Determination of the Hemolysis and Methemoglobin (MetHb) Percentage

RBCs (3% hematocrit) were incubated in Hepes buffer (25mM), pH 7.4, in the presence and absence of EC (0–25–50–75–100 μM), for up to 12 h. Subsequently, the change in the percentage of hemolysis was evaluated to determine the integrity of the membrane. The study was conducted through a spectrophotometric analysis, using the Beckman spectrophotometer DU 640 (Harbor Boulevard, Fullerton, CA, USA). In detail, readings were taken at 576 nm considering the ratio between the Hb released by erythrocytes and the total Hb contained by them. At the end of the analysis, the hemolysis levels were calculated using the following formula:

(1) H(%)=(ABSsample−ABS0)/(ABS100−ABS0)×100

where H (%) is the percentage of hemolysis reached; AbS_0_ the absorbance in the absence of EC; Abs_sample_ the absorbance of the samples in the absence or presence of EC; and AbS_100_ the absorbance of the sample at 100% hemolysis induced with water.

After the pellet was obtained with the different concentrations of epicatechin, it was lysed with ultrapure water and used to determine the possible production of methemoglobin, as described by Russo et al. [11].

Briefly, the red blood cells following the washes (3 times with NaCl 0.9%) were incubated with EC (0–25–50–75–100 μM) for 12 h. At the end of the incubation time, the EC-treated and non-EC-treated samples were centrifuged at 3000 rpm for 5 min, the supernatant was discarded, and the pellet was lysed with cold H_2_O (1:1 *v*/*v*). After centrifugation (18,000 rpm for 10 min), the percentage of MetHb was recorded in the spectrophotometer in the range of 500–680 nm.

### 4.4. Heat-Induced Hemolysis

RBCs, following washing three times with NaCl 0.9%, were pretreated in the presence and absence of epicatechin (50 and 100 μM), with 3% hematocrit, for 30 min. Subsequently, the samples were incubated at different temperatures (37 °C, 45 °C, 50 °C, and 55 °C) for 30 min. At the end of the incubation time, the samples were centrifuged at 3000 rpm for 5 min, and the supernatant was read by spectrophotometric analysis at 576 nm. Total hemolysis was assessed by lysing the red blood cells with cold H_2_O [48].

### 4.5. Evaluation of Osmotic Fragility

Osmotic fragility was assessed as reported by Naparlo K et al., with some modifications [49]. Briefly, following washing, the erythrocytes were resuspended with 10% hematocrit in Hepes buffer (pH 7.4) and incubated in the presence and absence of epicatechin (50 μM) for 5 and 90 min at 37 °C. Subsequently, 50 μL of the solution was withdrawn and brought to a volume of 1 mL with NaCl at different concentrations (0.34; 0.36; 0.38; 0.40; 0.42; 0.44%). Control samples were prepared by taking 50 μL of 10% RBCs that were added to 950 μL of H_2_O (for total hemolysis calculation) and 950 μL of 0.9% NaCl (negative control). The samples were then immediately centrifuged (for 5 min at 3000 rpm, 4 °C), and the absorbance of the supernatant was evaluated through a spectrophotometric analysis at 576 nm.

### 4.6. UV-Visible Spectroscopy Titration

The potential interaction between EC and Hb was studied by UV-Visible spectroscopy. The stock solution of Hb was obtained following lysis of red blood cells with iced distilled H_2_O. In detail, the red blood cells were lysed and then centrifuged at 20,000 rpm for 8 min. The pellet was discarded, while the supernatant was passed through a column of Sephadex G-25, balanced with Tris-HC1 0.01 M pH 8.0 buffer containing 0.1 M NaCl, and then through a column of Bio-Rad 501 × 8 mixed-bed ion exchange resin to remove organic phosphates and chloride ions. Finally, through a spectrophotometric analysis conducted at 576 nm, thus establishing the concentration of our stock solution, which was used to obtain the concentrations shown below.

Briefly, a solution of EC at known concentration (50 μM) was titrated by constant addition of Hb until a concentration of 12 μM was reached. The change in absorbances was analyzed in the range of 220–650 nm with a 1 cm optical path of the quartz cuvette. The experiment was repeated three times, and the data are presented as mean ± SD [50].

### 4.7. Effect of Hydrogen Peroxide on Erythrocyte Membrane Integrity

The RBCs, after the washes with NaCl 0.9%, were treated with EC (50 μM) and hydrogen peroxide (H_2_O_2_) (300 mM) to observe the potential protective effect of the molecule against stressogenic factors. Briefly, the samples were prepared with a 3% hematocrit in Hepes buffer (25 mM), pH 7.4, and pretreated with EC (50 μM) for 30 min, then centrifuged at 3000 rpm for 5 min and incubated with H_2_O_2_ (300 mM) for 2 and 14 h. Finally, the samples were centrifuged, and the supernatant was used for evaluation of the percentage of hemolysis. The negative control was exclusively RBCs in Hepes buffer, while the positive control was prepared by incubating the erythrocytes with H_2_O_2_ (300 mM) [51]. Hemolysis was measured by a spectrophotometric evaluation of the absorbance of the supernatant at 576 nm.

### 4.8. Morphological Analysis of the Erythrocyte Membrane

Following washing with NaCl 0.9%, the RBCs were resuspended in Hepes buffer (pH 7.4), hematocrit of 3%, and incubated with epicatechin (50 μM) for 30 min at 37 °C. Subsequently, the samples were centrifuged at 3000 rpm for 5 min, and the supernatant was discarded, while the pellet was resuspended (hematocrit 3%) in Hepes buffer (25mM), pH 7.4, and treated with hydrogen peroxide (H_2_O_2_) (50 mM) for 6 h at 37 °C. Subsequently, the samples were centrifuged, and the pellet has been diluted with Hepes buffer 25 mM (1:1; hematocrit at 1.5%) and used for morphological analysis through fluorescent microscope (with 100× objective) (Olympus BX60, Oaza-Odakura-Aza-Okamiyama, Japan). The negative control was prepared in the absence of the extract and in the presence of H_2_O_2_, while the positive control was prepared in the absence of H_2_O_2_ and in the presence of extract [51].

### 4.9. Flow Cytometry

The study was conducted with the FACS Canto II cytofluorometer (Becton Dickinson; software v8.0.3 BD FACSDiva™). Samples were analyzed with a gate physical side scatter (SS) versus forward scatter (FS) using a logarithmic scale. The readout was performed by histogram representation (using a linear scale) and/or by two-dimensional plot representation (cytogram dotblot). Briefly, erythrocytes were incubated overnight in the absence and presence of epicatechin 50 μM; subsequently, the cells were washed in 0.14 M NaCl, 0.01 M HEPES NaOH (pH 7.4) and 2.5 mM CaCl_2_ buffer (annexin-binding buffer). After being suspended in buffer, the RBCs were diluted 1:5 (0.5 % hematocrit) and subsequently, after the addition of annexin V (20 μL of stock solution), were incubated in the dark for 15 min and subsequently used for cytofluorometric analysis. Annexin fluorescence was measured through the FL-1 fluorescence channel, by direct dispersion analysis. The excitation and emission wavelengths were 488 nm and 530 nm, respectively.

### 4.10. Kinetic Measurements

Red blood cells were suspended in the incubation buffer (35 mM Na_2_SO_4_, 90 mM NaCl, 25 mM HEPES buffer, and 1.5 mM MgCl_2_), pH 7.4 at 25 °C and incubated with different concentrations of epicatechin (50 μM) [52]. The experiment was conducted at room temperature, 25 °C, and 37 °C to evaluate the effect of epicatechin at different temperature ranges. Following different time intervals (5, 15, 30, 60, 90 and 120 min), 10 μM of 4-acetamide-40-isothiocyanostilbene-2,20-disulfonic acid (SITS) was added to the reaction mixture to stop the reaction. The RBCs were centrifuged at 4000 rpm for 10 min to remove the working buffer and then washed three times with NaCl (0.9%). Subsequently, the erythrocytes were treated with perchloric acid (4%) and lysed with ultrapure H_2_O and recentrifuged. A solution containing glycerol and distilled water (1:1, *v*/*v*), 4 M NaCl, 1 M HCl and 1.23 M BaCl_2_⋅2H_2_O was added to remove sulphate ions and thus obtain a homogeneous precipitate of barium sulphate. Supernatant absorbance was evaluated in the range of 350–425 nm [53]. The values on the concentration of sulphate over time were obtained through the following equation:

c(t)=c∞(1−e−kt)

where c(t): concentration of sulphate at time t; c∞: intracellular concentration of sulphate at equilibrium; k: sulphate inflow rate constant.

### 4.11. Caspase 3 Assay

Caspase activity was assessed as reported by Galtieri et al. [54]. Briefly, erythrocytes were suspended in a working buffer (35 mM Na_2_SO_4_, 90 mM NaCl, 25 mM HEPES, 1.5 mM MgCl_2_), at pH 7.4 and incubated for 30 min at 37 °C in the presence and absence of epicatechin (50 μM). To obtain a positive control, we used tert-butyl-hydroperoxide (t-BHT) 100 μM, a known caspase activator. Afterwards, the samples were centrifuged, and the supernatant was discarded, while the pellet was suspended in Hepes buffer (100 mM) pH 7.4, lysed by sonication, and recentrifuged at 15,000 rpm for 10 min. The supernatant obtained was filtered through a Microcon YM 30, and then incubated for 1 h at 37 °C with the specific substrate of the caspase 3 AcDEVD-pNA and adjusted in volume with a buffer. The release of pNA was analyzed through a spectrophotometric analysis at 405 nm.

### 4.12. ATP Measurement

#### 4.12.1. Measurement of Intracellular ATP

The concentration of intracellular ATP was evaluated by the luciferin-luciferase method, as reported by Tellone et al. [55]. Briefly, red blood cells in the presence and absence of EC (50 and 100 μM) were diluted with buffer (35 mM Na_2_SO_4_, 90 mM NaCl, 25 mM HEPES, 1.5 mM MgCl_2_) and incubated for 30 min at 37 °C with Mastoparan 7 (Mas 7), an inducer of Gi proteins. Subsequently, to block the reaction, the erythrocytes were treated with trichloracetic acid (TCA, 15%) and centrifuged at 3000 rpm for 5 min at 4 °C. The supernatant was discarded and stored, while the pellets were diluted with a solution of D-luciferin and Firefly lantern extract (FLE 250) at a ratio of 1:1. The light emitted was measured with a Bio Orbit 1251 luminometer (Bio-Orbit Oy, Turku, Finland).

#### 4.12.2. Measurement of Extracellular ATP

Supernatant was used to determine extracellular ATP, as reported by Russo et al. [11]. The red blood cells at the end of the incubation time (protocol used in the previous paragraph) were centrifuged, and the supernatant was collected. Next, 10 μL of the supernatant was diluted with distilled H_2_O (990 μL). Finally, 100 μL of the solution was diluted with D-luciferin and Firefly Lantern (FLE 250) (Sigma-Aldrich, St. Louis, MO, USA) in a ratio of 1:1. The emitted light was recorded with a Bio Orbit 1251 luminometer.

### 4.13. Determination of PTP-1B Activity

The influence of epicatechin on the phosphorylation state of Band 3 protein was evaluated by assay of phosphatase activity, as reported by Maccaglia et al., with some modifications. Erythrocytes treated and untreated with epicatechin (0–25–50–75–100 μM) were compared with samples incubated with orthovanadate (OV) 3 mM, a phosphatase inhibitor. The erythrocytes were lysed with an iced isotonic buffer (Tris 5 mM, KCl 5 mM) and subsequently centrifuged at 20,000 rpm for 8 min. Subsequently, the supernatant was discarded, and the pellet was resuspended in an isotonic buffer and recentrifuged. The operation was repeated until the membranes were cleaned. After red blood cell membranes, in the presence and absence of epicatechin, were resuspended in 25 mM of HEPES buffer containing 0.1 mM phenylmethanesulfonylfluoride (PMSF), 20 mM MgCl_2_, and 15 mM p-NPP and incubated at 37 °C for 60 min, the release of p-nitrophenol was detected at 410 nm [55,56].

### 4.14. Statistical Analysis

The data are expressed as means ± standard error of the means (SEM) and were statistically evaluated for differences using one-way or two-way analysis of variance (ANOVA), followed by the Tukey–Kramer test (SigmaPlot Version 12.0, Systat Software, San Jose, CA, USA). *p*-values less than or equal to 0.05 were considered significant.

## 5. Conclusions

The described research contributes to filling some of the gaps in our knowledge about the effects of dietary polyphenols and RBC health and metabolic state. Our results might constitute an important step to potential use of the polyphenol as a therapeutic agent to prevent and/or limit the development of diseases. Future studies could help further clarify the therapeutic potential of this flavanol.

## Data Availability

The data that support the findings of this study are available from the corresponding author upon reasonable request.
